# A new approach of nonparametric estimation of incidence and lifetime risk based on birth rates and incident events

**DOI:** 10.1186/1471-2288-7-53

**Published:** 2007-12-20

**Authors:** Henrik Støvring, Mei-Cheng Wang

**Affiliations:** 1 Research Unit for General Practice, University of Southern Denmark, J.B. Winsløwsvej 9A, 5000 Odense C, Denmark; 2 Department of Biostatistics, Johns Hopkins University, 615 N. Wolfe Street, Baltimore, MD 21205, USA

## Abstract

**Background:**

Incidence and lifetime risk of diabetes are important public health measures. Traditionally, nonparametric estimates are obtained from survey data by means of a Nelson-Aalen estimator which requires data information on both incident events and risk sets from the entire cohort. Such data information is rarely available in real studies.

**Methods:**

We compare two different approaches for obtaining nonparametric estimates of age-specific incidence and lifetime risk with emphasis on required assumptions. The first and novel approach only considers incident cases occurring within a fixed time window–we have termed this *cohort-of-cases *data–which is linked explicitly to the birth process in the past. The second approach is the usual Nelson-Aalen estimate which requires knowledge on observed time at risk for the entire cohort and their incident events. Both approaches are used on data on anti-diabetic medications obtained from Odense Pharmacoepidemiological Database, which covers a population of approximately 470,000 over the period 1993–2003. For both methods we investigate if and how incidence rates can be projected.

**Results:**

Both the new and standard method yield similar sigmoidal shaped estimates of the cumulative distribution function of age-specific incidence. The Nelson-Aalen estimator gives somewhat higher estimates of lifetime risk (15.65% (15.14%; 16.16%) for females, and 17.91% (17.38%; 18.44%) for males) than the estimate based on cohort-of-cases data (13.77% (13.74%; 13.81%) for females, 15.61% (15.58%; 15.65%) for males). Accordingly the projected incidence rates are higher based on the Nelson-Aalen estimate–also too high when compared to observed rates. In contrast, the cohort-of-cases approach gives projections that fit observed rates better.

**Conclusion:**

The developed methodology for analysis of cohort-of-cases data has potential to become a cost-effective alternative to a traditional survey based study of incidence. To allow more general use of the methodology, more research is needed on how to relax stationarity assumptions.

## Background

Diabetes is a severe disease, which is becoming increasingly prevalent in countries throughout the world [1-6]. From a public health perspective it is vital to get good estimates of the present and future burden of diabetes. One measure of primary interest is diabetes incidence, both with respect to calendar time and age [7]. If combined with a model for mortality, it allows estimating lifetime risk of diabetes, another important public health measure [8]. Also, if combined with data on birth rates, it is possible to obtain a projection of future incidence, often needed for planning of health care services.

As the annual risk of developing diabetes is low in a general population, only very few follow-up studies exist on a general population level. Alternatively, various types of surveys have been conducted [8,9], which have then been analyzed to estimate age-specific incidence rates. Obviously, subjects of different ages in a survey originate from different birth cohorts, but this has received little attention in this context. As a consequence, the life-time risk estimated from such approaches pertains to a hypothetical cohort subjected to the current age-specific incidence and mortality rates. Likewise, future incidence is predicted from assuming birth cohorts of a given size and then subject these to the same age-specific incidence and mortality rates observed in the survey.

In this paper we propose a different approach which from the outset links past birth rates to the occurrence of incident events in a (often relatively short) time window. We will term this type of data *cohort-of-cases data *as it is a cohort consisting entirely of cases. More specifically, we require the sample to include all subjects who have advanced to a certain end-point (failure event) within a given calendar time period–and only these cases. Further, we assume that the time origin (initiating event, birth time) of each case can be retrospectively identified. So far, statistical methods for this type of doubly truncated data have not (to the extent of the authors' knowledge) been extensively studied, when the rate of initiating events is not assumed constant over calendar time.

It should be noted that cohort-of-cases data are different than case-cohort data (see for example [10], where the phrase case-cohort was coined) as the latter refers to a study comparing cases to a random sample from the corresponding cohort. In contrast, the cohort-of-cases data studied here comprises a study population consisting only of cases, but possibly supplemented with additional information on the process of initiating events. Cohort-of-cases designs–in this sense–are generally considered efficient, in particular for diseases with a low rate of occurrence; see [11-15], and references therein. We also want to point out that cohort-of-cases data provide information different from the information of the cases in the case-cohort studies, although the two types of data do share common characteristics. As pointed out in ([10], p4), the failure time in case-cohort studies is usually defined as time from the beginning of follow-up to a failure event, whereas the failure time in cohort-of-cases is time from initiating event to failure event.

To illustrate how the model can be applied we will use data from Odense Pharmaco-Epidemiological Database (OPED). Briefly, this database contains information on all redemptions of medications prescribed by a physician and subsidized by the national health insurance at any pharmacy within in a well defined geographical area holding nearly 500,000 inhabitants. The drug class of interest here is that used to treat diabetes. While such data by definition only concern pharmacologically treated diabetes, they do offer the opportunity for comparing the proposed approach with the traditional approach–the main purpose of the present paper.

The paper is organized as follows. We first describe the data, both on births and incident events. We then introduce a methodology which yields a non-parametric maximum likelihood estimate of the age-specific incidence distribution based solely on cohort-of-cases data, possibly supplemented with a known birth rate. The non-parametric method does not directly provide measures of the uncertainty of the estimate, and so we propose a bootstrap method for obtaining measures of this uncertainty. We then briefly outline the traditional analysis, before we present and compare results when applying the two methods to the data. We finally discuss implications in the last Section.

## Methods

### Cohort-of-cases data on anti-diabetic treatment

For the period 1992–2003 the Odense Pharmaco-epidemiological Database (OPED) contains subject specific information on all prescriptions for subsidized medications redeemed at any pharmacy in the County of Fyn, as well as information on births, deaths and migration into and out of the County of Fyn. The tracking of individuals is based on the Civil Registration Number (CRN) which is assigned to all at birth or first immigration into Denmark, and which uniquely identifies all residents of Denmark. For each individual we identified all prescriptions of anti-diabetic agents in OPED. The anti-diabetic drugs are characterized by the first three characters of the so-called ATC-code being A10 [16]. We will not distinguish between the various types of anti-diabetic treatments, such as for example insulin (A10A) and oral anti-diabetics (A10B). Incident events are defined to be the first treatment event observed in the time window for subjects who did not have any previous events during a one year run-in period. The run-in period was either started at the start of the database or at the time of first immigration into Fyn of the subject, if the subject immigrated into Fyn during the observation period. Note, that this may well introduce a calendar-time-dependent misclassification and hence bias [17], but this will be ignored in the following as we are not studying secular trends in incidence. Also note, that by definition, these data will only allow us to study incidence of pharmacologically treated diabetes. We will use the words "treated" and "diseased" interchangeably, and ask the reader to keep in mind that the present analysis only pertains to pharmacologically treated diabetes.

### Birth rates

For the period 1891–2003, available data from Statistics Denmark were used to determine annual, national birth counts for each gender. To estimate the number of births within the county of Fyn, data was obtained on population size for Denmark as a whole, as well as for Fyn with the objective of rescaling. Population counts were available roughly at five year intervals (1901, 1906, ..., 1921, 1925, 1930, ..., 1970, 1976, 1981, 1986, 1990, 1995, 1998, 1999, ..., 2003) for Fyn, whereas nationwide data was available annually from 1970 and onward, and otherwise similar to those for Fyn given above. Only from 1970 can all members of a given birth cohort be followed up individually, and hence we only rely on annual counts that are available throughout.

**Figure 1:**
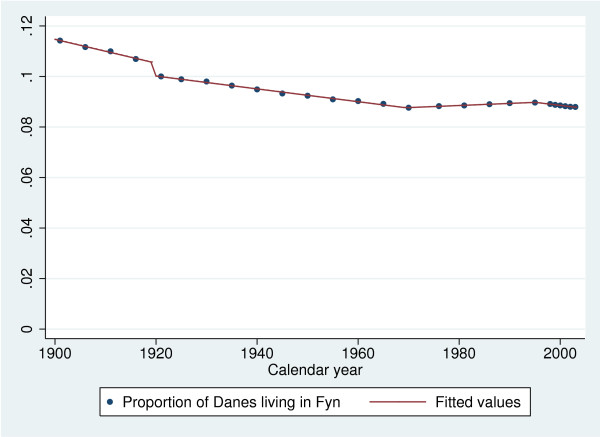
Observed and predicted fractions of the Danish population living in the county of Fyn during 1900–2003.

To estimate the number of births in the county of Fyn, we scaled national birthrates by the relative population size in the county of Fyn compared to the total population of Denmark. The underlying assumption is that the fertility rate on Fyn is similar to national rates, which seems plausible given the small size of Denmark and the relatively homogeneous composition of the population. As population counts are not available annually we interpolated the population data based on piecewise linear regression with cut points at 1920, 1970, and 1996, cf. Figure 1.

Overall, Fyn hold 9%–10% of the Danish population during most of the twentieth century and the fit seems very good. The sudden drop in 1920 is due to the reunion of North Slesvig with Denmark after having been part of Germany from 1864.

**Figure 2:**
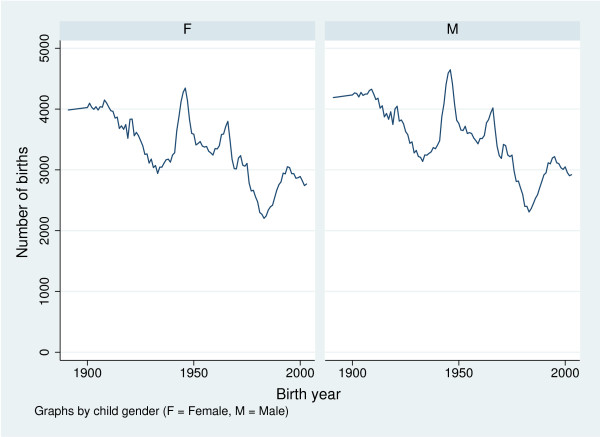
Annual number of births in the county of Fyn during 1891–2003.

In subsequent analyses the missing proportions were replaced with the predicted, while the observed proportions were retained. When we combined this with the national birth rates, we could compute the number of births in the county of Fyn as the product of the number of births in Denmark and the proportion of the Danish population living in the County of Fyn. Since no observations were available for the ten year period 1891–1900, we predicted the annual number of births in this period from a linear extrapolation of the birth counts in the period 1901–1910. The resulting gender specific annual birth rates in the County of Fyns are presented in Figure 2.

For the birth rates to be of value, we must assume that migration balances in the sense that immigration and emigration for each birth cohort prior to and within the observation window is expected to be of similar size. This is, however, reasonable in the present context as the relative size of the studied population is nearly constant compared to the entire Danish population. In all subsequent analyses, estimated numbers of births are treated as fixed.

### Methodological set-up

Let us now introduce the notation used in the paper. Let *U *be the calendar time of the initiating events (births). Let *Y *be age at onset if disease occurs before death, and infinity in the absence of disease before death. Let the probability density function (pdf) of *Y *be *f*(*y*|*u*), and the associated cumulative distribution function (cdf) *F*(*y*|*u*). Further, let *Z*_0 _be age at death if *Z*_0 _<*Y*, that is disease does not occur before death. If *Y *> *Z*_0_, we let *Y *= ∞, and otherwise we let *Z*_0 _= ∞. To avoid ambiguity, we will at times denote *F *as *F*_*Y*_.

Since not all subjects will experience disease prior to death, the pdf of *Y*, *f*(*y*|*u*), is a mixture distribution with two components:

*f*(*y*|*u*) = *π*_∞ _(*u*)f*(*y*|*u*) + (1 - *π*_∞ _(*u*))*I*(*y *= ∞)

where *π*_∞ _(*u*) is defined as *P *(*Y *< ∞ |*u*), i.e., it is the probability of disease occurring before death, *I*(·) is an indicator function, and f*(*y*|*u*) is the conditional pdf of *Y *given that *Y *< ∞, i.e., *Y *≤ *Z*_0_. Note, that since *π*_∞ _(*u*) is the probability of disease occurring before death for a subject with birth at *u*, it is the lifetime risk for subjects with birth time *u*.

#### Cohort-of-cases data

**Figure 3:**
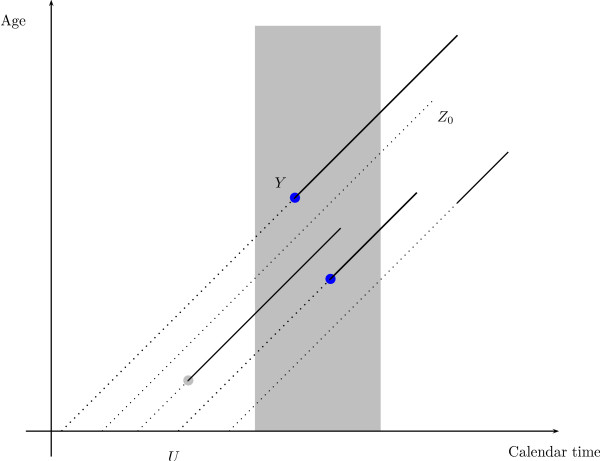
Lexis diagram with observation window (gray area). Dotted lines indicate lifetime without disease until age of onset (*Y*), or age at death (*Z*_0_), full lines lifetime with disease. Only age at onset times within the observation window are observed (blue points) in a cohort-of-cases study.

Assume that we observe all ages of onset, *Y*, occurring within the calendar time observation window [0; *τ*_0_), cf. Figure 3 for a graphical presentation of the sampling scheme. Assume that the occurrence of births follows a Poisson process with intensity *φ*(*u*) for *u *≤ *τ*_0_, and that *y*^+ ^= sup{*y*: F* (*y*|*u*) < 1} exists and is finite for all *u *≤ *τ*_0_, i.e., *y*^+ ^is the maximal observable age at onset before death. We can then normalize the birth intensity *φ*(*u*) to a density *g *on [-*y*^+^; *τ*_0_),

$$g(u)=\frac{\phi (u)}{\int_{-y^{+}}^{\tau_{0}}\phi (s)ds}$$

with associated cumulative distribution function *G*. In principle *φ *could well depend on covariates, but since we consider *φ *either known or constant, we will ignore this.

We will in the following assume (*U*_1_, *Y*_1_),..., (*U*_*n*_, *Y*_*n*_) to be independent and identically distributed (iid). Two crucial assumptions must be considered. First, whether or not we have calendar time stationarity with respect to age of onset, i.e.,

**(S1) **Age of onset is independent of time of birth, i.e., *F*(*y*|*u*) = *F*(*y*).

Secondly, knowledge about the birth process will not be available in many applications. Hence we also consider the situation with calendar time stationarity of the birth process:

**(S2) **Assume that the occurrence of initiating events, births, started in the distant past and that this birth rate has been stabilized. Or, quantitatively, assume that *u*_*x *_= inf{*u*: *φ*(*u*) > 0} is small enough so that *u*_*x *_≤ -*y*^+^, and that *g *is uniform on [-*y*^+^; *τ*_0_).

#### Stationary incidence, known birth process ((S1) only)

When only (S1) holds, the joint density of the observed (*u*, *y*) can be written as follows:

$$\begin{array}{lll} p(u,y|-U\leq Y\leq \tau_{0}-U) & = & \left[\frac{g(u)I(-y\leq u\leq \tau_{0}-y)}{G(\tau_{0}-y)-G(-y)}\right]\\ & & \times \left[\frac{\left\{G(\tau_{0}-y)-G(-y)\}f^{*}(y)I(y\leq y^{+}\right)}{\int_{0}^{y^{+}}\{G(\tau_{0}-s)-G(-s)\}f^{*}(s)ds}\right] \end{array}$$

≡ *p*_*c*_(*u*|*y*)*p*_*m*_(*y*)

where *p*_*c*_(*u*|*y*) and *p*_*m*_(*y*) are defined by the expressions in each bracket in (3), respectively. Thus *p*_*c*_(*u*|*y*) can be interpreted as the density of birth times conditional on *y *being observed in [0; *τ*_0_), and *p*_*m*_(*y*) as the marginal density for the observed *y *weighted with *w*_*i *_= *G*(*τ*_0 _-*y*) - *G*(-*y*), i.e., the probability of birth occurring within the interval [-*y*; *τ*_0 _-*y*).

When *g *is known, then so is *p*_*c*_, as are the weights in *p*_*m*_. It is thus straightforward to compute the maximum likelihood estimate of F* based on the weighted observations:

$${\hat{F}}^{*}(y)=\frac{\sum_{i:y_{i}\leq y}w_{i}^{-1}}{\sum_{i=1}^{n}w_{i}^{-1}}$$

The estimate thus places mass $w_{j}^{-1}/\sum w_{j}^{-1}$ at each jump point *j*, where *j *corresponds to the observation number in the ordered set of *Y*_*i*_. That the estimate in (5) is the non-parametric maximum likelihood estimate (NPMLE) follows directly from standard results on NPMLE, see for example the paper by Turnbull [18], who covers the general case of which this is a special case. If all weights are equal, the above formula reduces to the ordinary formula for non-parametric estimation of a cdf in the uncensored case, putting mass *n*^-1 ^at each jump point.

With the estimate of the conditional cdf F* it is possible to obtain an estimator of the unconditional *F *utilizing their relationship given in Equation (1). What we need is an estimate of *π*_∞_, which may be obtained from noting that the occurrence rate of incident events, *I*^tr^, at any calendar time point is given by

$$I^{\text{tr}}(t)=\int_{-\infty }^{t}\phi (u)f(t-u)I(t-u\leq y^{+})du$$

$$=\pi_{\infty }\int_{-\infty }^{t}\phi (u)f^{*}(t-u)du$$

where the indicator function *I*(*t *- *u *≤ *y*^+^) is needed, since the occurrence rate does not include those for which onset never happens, that is when *y *= *t *- *u *> *y*^+^or equivalently that *y *= *t *- *u *= ∞. Integrating this over the observation window, we find

$$\int_{0}^{\tau_{0}}I^{\text{tr}}(t)dt=\pi_{\infty }\int_{0}^{\tau_{0}}\int_{-\infty }^{t}\phi (u)f^{*}(t-u)du\;dt$$

$$=\pi_{\infty }\int_{-\infty }^{\tau_{0}}\phi (u)\left\{\int_{\max(u,0)}^{\tau_{0}}f^{*}(t-u)dt\right\}du$$

$$=\pi_{\infty }\int_{-\infty }^{\tau_{0}}\phi (u)\left\{\int_{\max(0,-u)}^{\tau_{0}-u}f^{*}(t)dt\right\}du$$

$$=\pi_{\infty }\int_{-\infty }^{\tau_{0}}\phi (u)\left\{F^{*}(\tau_{0}-u)-F^{*}(\max(0,-u))\right\}du$$

from which it follows that

$$\pi_{\infty }=\int_{0}^{\tau_{0}}I^{\text{tr}}(t)dt/\left[\int_{-\infty }^{\tau_{0}}\phi (u)\left\{F^{*}(\tau_{0}-u)-F^{*}(\max(0,-u))\right\}du\right]$$

Although the estimate is intuitely attractive it is not clear whether it is the MLE. However, if we fill in the MLE of F* in Equation (12), we do obtain an estimate of *π*_∞_, since *φ *is known and $\int_{0}^{\tau_{0}}I^{\text{tr}}(t)dt$ is estimated by the total number of observed incidences over the interval [0; *τ*_0_). Having obtained the MLE of F* together with an estimate of *π*_∞_, we can use Equation (1) to compute an estimate of *F*, the unconditional cdf of age at onset.

#### Stationary incidence, stationary birth process (both (S1) and (S2))

When both (S1) and (S2) hold, the marginal density of the observed *y*'s can be further simplified by substitution of the uniform birth density in the corresponding expression in Equation (3), i.e.,

$$p_{m}(y)=\left[\frac{\left\{\tau_{0}/(\tau_{0}+y^{+})\}f^{*}(y)I(y\leq y^{+}\right)}{\tau_{0}/(\tau_{0}+y^{+})}\right]=f^{*}(y)I(y\leq y^{+})$$

Note, that here the density function of the observed *y*'s coincides with the population density function f* of the observable onset times, *Y *. In the case when only age at onset distribution is of interest, and not lifetime risk, the 'usual methods' are thus applicable to the case data to estimate f* by putting equal weights on all observations as noted above.

If, however, we are also interested in the unconditional density, *f*(*y*), we need an estimate of *π*_∞ _to be able to proceed. Above, this was obtained from our knowledge of the birth process, and in principle we could exploit this again. However, in situations where a stationary birth process is assumed, this is typically because we lack information on the birth process. Thus it may in such situations be necessary with alternative approaches. One obvious way to proceed is the following: In the time window where information is collected on incident cases, we also collect information on deaths–either for all or a random sample–and classify them according to whether or not they had experienced disease. The relative frequency of diseased deaths will then be an estimate of *π*_∞ _under stationarity assumptions with respect to the birth process, the incidence process, and the mortality. This estimate is valid if age-specific mortality is assumed stationary both among diseased and non-diseased–these strong assumptions reflect the lack of available information in such situations. With this estimate of *π*_∞ _we may then estimate the unconditional *F*.

#### Non-stationary incidence, known birth process (Neither (S1) nor (S2))

When neither (S1) nor (S2) hold, the likelihood becomes substantially more complicated. In principle, this can be handled by introducing a parameter vector *θ *which relates the incidence density to the time of birth. The rewriting presented in Equation (4) is still valid with the modification that the density term *p*_*m*_(*y*) now depends on the parameter vector *θ*, i.e.,

$$p_{m}(y|\theta )=\frac{\left\{G(\tau_{0}-y|\theta )-G(-y|\theta )\}f^{*}(y|\theta \right)}{\int_{0}^{y^{+}}\{G(\tau_{0}-s|\theta )-G(-s|\theta )\}f^{*}(s|\theta )ds}$$

Unfortunately this density does not directly permit use of the approach presented above for finding a non-parametric estimate of f*(*y*|*θ*), nor for finding the corresponding estimate of *π*_∞ _(*θ*).

One alternative is to set up a full likelihood by considering a full parametric model of both age of onset and age of death, but we will not go into further details here and instead commend this as a topic for future research.

### Ordinary non-parametric analysis

The ordinary Nelson-Aalen analysis based on observed events and time at risk is well described elsewhere, see [19] for an extensive treatment of the subject, or [20] for a more focused treatment. In short, we use age as the fundamental time scale, and we then have delayed entry due to the fact that not all subjects are followed from birth. Rather, they enter the observation window and capture area at a certain age and are then followed until either event or censoring, whichever comes first.

We let subjects become at-risk one year after the start of the observation period if they resided in Fyn County in this period, or one year after entrance to the capture area, if they immigrated to Fyn during the study period. In both cases the one year run-in period is used to identify subjects not already in treatment (those without filled prescriptions in the period), as only they are at risk for becoming incident. Subjects cease to be under observation either at onset, death, emigration from Fyn, or end of follow-up, whichever comes first.

As above we require calendar time stationarity for estimation of *F*. The second assumption in this setup is that entry is independent of disease onset, i.e., age at immigration to Fyn County is not informative for the subsequent distribution of *Y*. The final assumption is that censoring is non-informative. The two latter assumptions are similar, but not identical, to the assumption of balance of migration made in the analysis of doubly truncated data. The difference is, that independent delayed entry and censoring only concerns the time within the observation period. On the other hand, the balancing assumptions does not require independence, i.e., migrating subjects may well have a different morbidity than non-migrating subjects–which is indeed the case [21]–as long as the distribution of onset ages is similar among immigrating and emigrating subjects.

Thus we get a non-parametric estimate of Λ_*Y*_, the cumulative hazard for onset of disease. Similarly, a non-parametric estimate of the cumulative hazard of death among non-diseased, $A_{z_{0}}$, can be obtained by simply exchanging the event indicator from onset of disease to death and maintaining the at-risk time.

From Λ_*Y *_and $A_{z_{0}}$ an estimate of *F *is given by (cf. [22] for a theoretical discussion, while [23] gives an example of its application)

$$\begin{array}{ccc} F(y) & = & \int_{0}^{y}\lambda_{Y}(s)\exp[-\Lambda_{Y}(s)]\exp[-A_{z_{0}}(s)]ds\\ & & +I(y=y^{+})(1-\pi_{\infty }) \end{array}$$

where *λ*_*Y *_is the hazard associated with Λ_*Y*_, and *π*_∞ _is the lifetime risk given by

$$\begin{array}{ccc} \pi_{\infty } & = & \int_{0}^{y^{+}}\lambda_{Y}(s)\exp[-\Lambda_{Y}(s)]\exp[-A_{z_{0}}(s)]ds \end{array}$$

As no analytic confidence intervals are available for the lifetime risk, we obtained them using bootstrap as above. This can also be applied to obtain age-specific confidence intervals for *F*.

### Projection of incidence

Based on an estimate of *F*, projection of incidence is possible both inside and outside the observation window by application of the formula in Equation (6), when the birth process is known and incidence is assumed stationary. In the application studied here, the birth process is known for *u *≤ *τ*_0_. For *u *> *τ*_0 _it must be projected. Hence, we carry the last observed value of the birth process forward, i.e., let *φ *(*u*) = *φ *(*τ*_0_) for *u *> *τ*_0_.

## Results and Discussion

Table 1 gives basic descriptive statististics of the studied population, as it shows the number of incidence events tabulated by gender, birth period and calendar year, which is used for estimating age-specific incidence.

**Table 1:** Number of incidence events by gender, calendar year of event and calendar year of birth

		Event year										
Gender	Birth	1993	1994	1995	1996	1997	1998	1999	2000	2001	2002	2003
Females	-1909	60	24	30	22	7	19	9	7	6	4	4
	1910-9	103	79	74	101	81	69	66	58	65	51	47
	1920-9	110	123	99	122	98	112	118	109	106	119	118
	1930-9	65	86	78	86	96	82	96	108	111	132	140
	1940-9	51	51	55	70	65	64	88	100	115	117	137
	1950-9	41	31	32	26	32	30	38	48	51	64	70
	1960-9	18	18	13	20	8	18	28	29	30	34	48
	1970-9	17	10	9	6	13	5	9	9	18	19	34
	1980-9	4		3	24	5	6	8	7	9	5	11
	1990-				4	6	3	3	5	7	10	10

Males	-1909	29	19	18	8	7	7	4		2	2	2
	1910-9	94	80	68	71	65	58	42	45	37	21	28
	1920-9	126	145	106	118	96	116	99	93	82	123	123
	1930-9	107	95	114	132	126	119	131	156	143	126	174
	1940-9	104	102	83	102	111	129	140	166	183	191	214
	1950-9	49	52	38	52	42	77	65	70	77	106	113
	1960-9	16	19	19	21	27	23	28	29	41	55	53
	1970-9	12	11	17	8	5	7	10	9	15	14	12
	1980-9	8	2	3	28	6	2	7	12	12	12	10
	1990-				5	3	2	6	7	8	9	5

Number of incidence events by gender, calendar year of event and calender year of birth.

### Cohort-of-cases data

#### Complete stationarity

**Figure 4:**
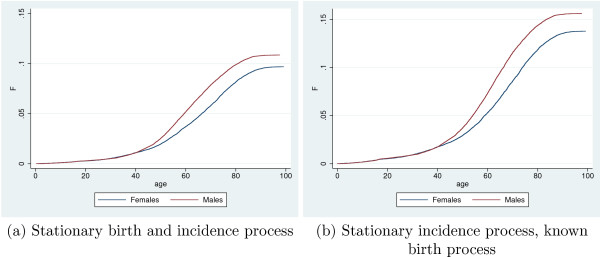
Estimated incidence distribution *F *for pharmacological treatment with any anti-diabetic drug with respect to age, and stratified on gender.

Although the birth process is known in our setting, we for comparison present an analysis based on assuming stationarity for the birth process, the incidence process, as well as the mortality process among treated. We first classified all deaths according to whether or not a previous redemption of anti-diabetics had been observed, considering all with such a redemption to be diabetics. The lifetime risk, *π*_∞_, was for females estimated at 9.68% (95% Confidence Interval: 9.35%; 10.02%) and for males at 10.86% (10.51%; 11.22%), where both confidence intervals are binomial exact. The estimated incidence distribution, *F*, stratified on gender is shown in Figure 4(a).

#### Stationarity of incidence, known birth process

When only stationarity of the incidence distribution is assumed, a non-parametric analysis based on the weighted likelihood given in Equation (4) and the estimator of *π*_∞ _in Equation (12) can be conducted. With the gender specific birth rates, we estimated gender specific estimates of F*, *π*_∞_, and hence *F*, from the observed events and associated ages at the events. The resulting estimates of the incidence distribution *F *are displayed in Figure 4(b).

We see that the incidence distribution for both genders are made up of two components: The first component is a more or less constant density for ages below 40 years (the linear part in *F*), whereas the second is a much higher, unimodal density for ages above 40 years which vanishes for ages above 80 (the sigmoid shaped part of *F*). For females the lifetime risk, *π*_∞_, was estimated at 13.77% (13.74%; 13.81%), for males at 15.61% (15.58%; 15.65%). Both confidence intervals are computed using bootstrap with a thousand replications. The confidence intervals are very narrow which reflects the high statistical efficiency of the weighted likelihood approach–which in turn partly comes from the strong assumption of stationarity. As birth counts are assumed known, this too contributes to the narrow confidence intervals, although to a lesser degree.

The shape of *F *is quite similar to the unweighted estimate, whereas the estimated lifetime risks are substantially higher than those estimated above. The major explanation is of course lack of stationarity of the true lifetime risk and/or the disease duration: The estimate of *π*_∞ _based on disease status among observed deaths takes most of its information from the older cohorts as they are the ones with high mortality. If the older cohorts had lower lifetime risk and/or previously had relatively higher mortality among diseased compared to non-diseased (both of these scenarios are very realistic, but contrary to assumptions of the previous analysis), this will result in a decreased estimate of *π*_∞ _. This would be amplified if older cohorts are larger than younger cohorts, as is indeed the case here, cf. Figure 2.

Contrastingly, when indirectly estimating *π*_∞ _based on weighting with the birth process, the estimate can be viewed as a weighted average of *π*_∞ _over the entire interval for the birth process [-*y*+; *τ*_0 _-*y*).

#### Projection of diabetes incidence

**Figure 5:**
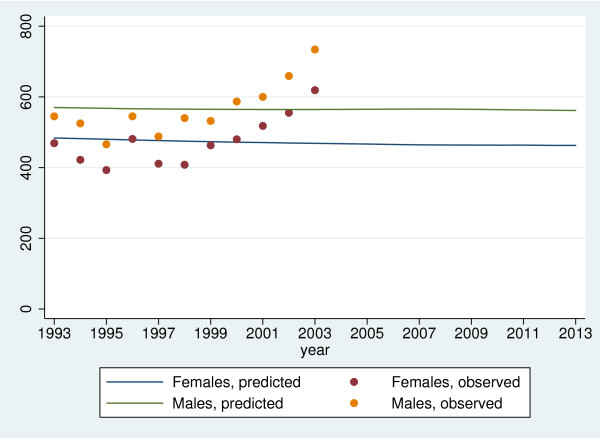
Projected and observed annual numbers of incident events in the county of Fyn based on an assumption of a stationary incidence and using a weighted, non-parametric estimate of *F*.

In the completely stationary situation, where (S1) and (S2) are both assumed to hold, the projected annual incidence is a constant number equaling the lifetime risk multiplied by the annual number of births. As the annual number of births are usually not observable in such settings, an alternative is needed. In the spirit of estimating *π*_∞ _from the treatment status among deaths, one could take the total annual number of deaths as an estimate of the number of births. The reasoning for this is that if the population is in a completely stationary state, the annual number of deaths must on average equal the average annual number of births. In our setting the observed numbers of deaths over the 11 year period are 29,871 for females and 29,816 for males yielding projected, annual incidences of 262.8 for females and 294.3 for males. In Figure 5 the incidence is projected based on the weighted, non-parametric estimate of *F *obtained above, i.e., with known birth intensity and stationary incidence. All annual birth counts after 2003 are set to the number of births observed in 2003. Note, that the observed incidence strongly suggests a departure from stationarity, and so future actual incidences are likely to be higher than those projected from a stationarity assumption. The projected incidences show a small but persistent decline for 2004–2013 due to declining birth rates in the last half of the twentieth century. The general level is much higher than above, reflecting the higher estimate of *π*_∞ _obtained from using the known birth distribution, but correspond well with observed incidences.

Ideally, projections should be accompanied by confidence intervals, but we have been unable to compute them. While in principle some variant of bootstrap might be employed, this is numerically very demanding as the entire cdf of age-specific incidence must be bootstrapped. Judged from the conifdence intervals of the lifetime risks, the confidence intervals of the projections will be very small, reflecting both high efficiency of the method, as well as its strong assumptions.

### Ordinary non-parametric analysis

**Figure 6:**
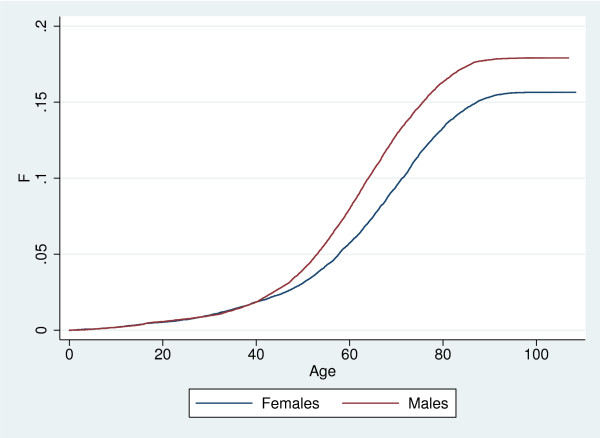
Estimated incidence distribution *F *for pharmacological treatment with any anti-diabetic drug with respect to age, and stratified on gender. Ordinary non-parametric estimate with independent delayed entry.

The gender specific estimates of *F *are shown in Figure 6. The shape of the estimated cdf is very similar to the one obtained above using a known birth process for weighting. The estimated lifetime risks are 15.65% (15.14; 16.16) for females, and 17.91% (17.38; 18.44) for males, where confidence intervals were found from bootstrap with 1,000 replications. This is somewhat higher than when analyzing data as doubly truncated. The explanation is that mortality has generally declined substantially over the past century, and hence an estimate based on the mortality rates observed within the observation window leads to a higher risk of diabetes onset prior to death than an estimate which implicitly accounts for past mortality.

#### Projection of diabetes incidence

**Figure 7:**
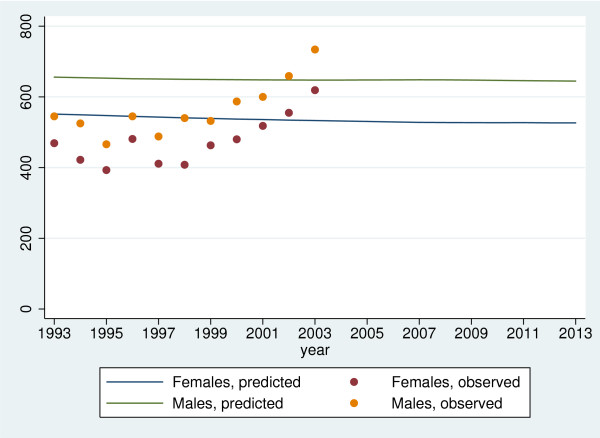
Projected and observed annual numbers of incident events in the county of Fyn based on the ordinary non-parametric estimate of *F *with independent delayed entry.

Projections are obtained as above–except that the ordinary non-parametric estimate of *F *is used–and results are shown in Figure 7. Due to the elevated lifetime risk the projected incidences are now higher–also too high compared to observed incidences. Also for this projection we have been unable to provide confidence intervals for the same reasons as above.

## Conclusion

In this paper we have developed and implemented methods for estimating and projecting incidence, as well as the lifetime risk of a disease based on observation of incident events in an observation window, i.e., what we termed cohort-of-cases data. The developed methodology yields non-parametric estimates comparable to those of a standard Nelson-Aalen analysis based on independent delayed entry, but it gives slightly better projections of incidence due to its implicit accounting for the unobserved mortality among untreated in the past.

In its simplest form–i.e., assuming both a stationary birth process and incidence–a simple non-parametric estimate of the age of onset distribution is obtained. When alternatively the birth process is considered known, this is taken into account by a weighted, non-parametric estimate with weights based on the relative sizes of the relevant birth cohorts. Both approaches directly provide estimates of age-specific incidence as well as of lifetime risk, which are of considerable public health interest. Due to the relatively fast computational procedures developed, confidence intervals for the lifetime risk could be obtained from direct application of bootstrap methodology. We were however unable to provide confidence intervals for projection of incidence.

As stated by Narayan *et al. *in 2003, lifetime risk of diabetes appears not to have been estimated prior to their paper [8], and only one subsequent paper have reported comparable estimates of lifetime risk [24]. The directly comparable estimates for the US population found in [8] are substantially higher (39% for females, 33% for males) than ours (14% for females, 16% for males). The two major reasons for the difference is a generally lower diabetes incidence in Denmark [4], as well as the fact that our estimates only pertain to pharmacologically treated diabetes. It would however be interesting to explore if part of the difference is due to their use of the traditional method, as the traditional method in our material leads to an elevated estimate of lifetime risk of 16% for females and 18% for males. It is further interesting that the gender differences are in opposite directions in the two countries.

Several papers have used estimates of incidence to project the future burden of diabetes, most prominently [2,5,6]. For all three, it would be interesting to re-analyze their data using our developed method for cohort-of-cases data, if possible, to see if a similar discrepancy exist between the two analytical methods as we have found, where the traditional method lead to an inflated projection of the number of incident events of diabetes, when compared to the observed count.

For the theoretical developments, assumptions (S1) and (S2) have been crucial, but from an applied perspective the assumptions are very restrictive. In our application concerning diabetes, the assumptions are likely not satisfied, as it is questionable that both age-specific incidence and age-specific mortality among diabetics have been constant since 1900–rather, changes in incidence due to altered lifestyle, and changes in mortality due to improved treatment and general health are reasonable. Indeed, it is known that within the observation window of 1993 and 2003, statistically significant trends exist for both quantities [4]. Yet the predictions based on the developed model are at least as good as those based on the ordinary non-parametric method, showing the potential of the developed model. More work on relaxing the assumptions is however mandated before the model can be used more generally.

Although we in principle showed how the stationarity assumption could be relaxed by formulating a full, parametric likelihood, we did not give a detailed analysis of this situation due to its complexity. Also, the data considered in this paper are rather limited since, first, the observation window is short compared to typical disease duration, and second, no information is available on age of onset outside the observation window. As a result, we have been unable to allow for trends in incidence and mortality, the absence of which must be considered unrealistic. In some epidemiological settings it will, however, be possible to obtain data on age of onset for subjects prevalent at start of the time window or for diseased subjects dying in the observation window [25]. While such information is valuable and needs to be incorporated in the analysis to allow relaxation of assumptions, it requires knowledge about the past mortality among diabetics. In contrast, we have tried to develop a methodology that only rely on observation of incident events and past birth rates, which are often easier to obtain. There is, however, a need for further research on the applicability and extensions of the method before its potential can be more clearly appreciated.

## Competing interests

The author(s) declare that they have no competing interests.

## Authors' contributions

HS had the original idea for the study, carried out all analyses and drafted the original and revised manuscripts with substantial input from MCW. The planning of analyses and interpretation of the data was the joint product of discussions between MCW and HS. Both authors have seen and approved the final version.

## Pre-publication history

The pre-publication history for this paper can be accessed here:


